# Sound reasons for unsound sleep: Comparative support for the sentinel hypothesis in industrial and nonindustrial groups

**DOI:** 10.1093/emph/eoac039

**Published:** 2022-11-22

**Authors:** Leela McKinnon, Eric C Shattuck, David R Samson

**Affiliations:** Department of Anthropology, University of Toronto Mississauga, Mississauga, ON, CanadaL5L 1C6; Department of Anthropology, University of Toronto Mississauga, Mississauga, ON, CanadaL5L 1C6; Institute for Health Disparities Research, University of Texas at San Antonio, San Antonio, TX 78249, USA; Department of Public Health, University of Texas at San Antonio, San Antonio, TX 78249, USA; Department of Anthropology, University of Toronto Mississauga, Mississauga, ON, CanadaL5L 1C6

**Keywords:** sleep, sentinel hypothesis, human evolution, evolutionary mismatch, health disparities

## Abstract

**Background and objectives:**

Sleep is a vulnerable state in which individuals are more susceptible to threat, which may have led to evolved mechanisms for increasing safety. The sentinel hypothesis proposes that brief awakenings during sleep may be a strategy for detecting and responding to environmental threats. Observations of sleep segmentation and group sentinelization in hunter-gatherer and small-scale communities support this hypothesis, but to date it has not been tested in comparisons with industrial populations characterized by more secure sleep environments.

**Methodology:**

Here, we compare wake after sleep onset (WASO), a quantitative measure of nighttime awakenings, between two nonindustrial and two industrial populations: Hadza hunter-gatherers (*n* = 33), Malagasy small-scale agriculturalists (*n* = 38), and Hispanic (*n* = 1,531) and non-Hispanic White (NHW) (*n* = 347) Americans. We compared nighttime awakenings between these groups using actigraphically-measured sleep data. We fit linear models to assess whether WASO varies across groups, controlling for sex and age.

**Results:**

We found that WASO varies significantly by group membership and is highest in Hadza (2.44 h) and Malagasy (1.93 h) and lowest in non-Hispanic Whites (0.69 h). Hispanics demonstrate intermediate WASO (0.86 h), which is significantly more than NHW participants. After performing supplementary analysis within the Hispanic sample, we found that WASO is significantly and positively associated with increased perception of neighborhood violence.

**Conclusions and implications:**

Consistent with principles central to evolutionary medicine, we propose that evolved mechanisms to increase vigilance during sleep may now be mismatched with relatively safer environments, and in part responsible for driving poor sleep health.

## INTRODUCTION

Sleep is a critical component of human health. Insufficient sleep has immediate effects on cognition and long-term negative consequences for metabolic, immune, and neurological function [1–3]. Chronic sleep insufficiency is linked to higher levels of C-reactive protein (CRP), interleukin-6 (IL-6), and white blood cell count, markers indicating systemic inflammation that are associated with numerous chronic diseases and overall mortality risk [4, 5]. Despite its benefits, sleep is a behaviorally vulnerable state that likely increased the sleeping individual’s risk of predation, conspecific violence, and exposure to temperature fluctuation and inclement weather, with potentially harmful outcomes over the course of human evolutionary history. Evolved behavioral strategies to offset this risk are demonstrated in numerous species that are vulnerable to predation including birds, rats, and non-human primates, where sleep duration, timing, and intensity are observed to change depending on perceived threat of predation [6]. For example, birds have been observed to scan their sleeping sites for predators by briefly opening their eyes periodically during sleep periods, a scanning behavior that is less prominent in larger groups which presumably afford more security through communal vigilance [7]. In addition, humans demonstrate sleep behavior that is thought to reflect evolutionary pressures that necessitated increased vigilance. Sleep segmentation (i.e. sleep divided into periods, with pronounced wakefulness during the night) and staggering of sleep periods in groups may increase vigilance throughout the night, which is a pattern that has been documented in small-scale subsistence societies [8, 9]. However, the extent to which these adaptive responses to potential threat persist and influence sleeping patterns in industrial contexts has not yet been thoroughly explored.

### Terrestrial sleep as a uniquely human behavior

Terrestrial sleep is unusual among primates. For chimpanzee (*Pan troglodytes*) populations where predation is low, a small proportion of males have been observed to ground-sleep [10]. Large non-human primates, including gorillas (*Gorilla gorilla*), are observed to sleep both arboreally and terrestrially, depending on predation risk. Physically massive male gorillas often sleep on the ground [11], while female and juvenile gorillas sleep terrestrially or arboreally depending on the presence of a protective silverback gorilla in the group [11]. Yet, of all the primates, only humans habitually sleep terrestrially across age and sex classes. Therefore, it is proposed that during the tree-to-ground transition that occurred around 2 million years ago, hominid sleep underwent profound changes as a result of fully terrestrial sleep; based on morphological changes to post-crania that became exclusively suited to terrestrial movement, these changes in sleep likely became defining human features by the emergence of *Homo erectus* [12, 13]. Despite the loss of safety afforded by arboreal sleeping, humans appear to have evolved mechanisms to minimize the risks inherent to this complete transition to the ground. The controlled use of fire would have offered protection from predators [14], as well as opportunities to augment social cohesion and learning through conversation [15]; yet, reliance on fire can also necessitate periods of awakening throughout the night for its maintenance.

The *sentinel hypothesis* proposes that the combination of sleeping asynchronously in groups coupled with brief, passive periods of awakening around rapid-eye movement (REM) sleep staging allow for environmental scans within a socially protected sleep site which maximizes the balance between sleep continuity and alertness for danger [16]. Alternation between NREM and REM sleep in human sleep cycles is proposed to serve a protective function, in which consecutive time in high arousal threshold sleep (i.e. “deep sleep”) is short, thereby minimizing time that danger can approach undetected. Deep sleep and REM sleep alternate with light sleep (and not with each other), and it is thought that the return to light sleep allows better signal detection. Awakenings typically associated with the ending of REM phases provide further protection with periodic screening of the environment [17]. Blume and colleagues [18] report that in their electroencephalogram study measuring processing of auditory stimuli during sleep, there is evidence for processing of voice familiarity through all NREM and even REM stages. They propose that during sleep, human brains *passively* enter a ‘sentinel processing mode,’ in which they are able to evaluate environmental stimuli [18]. Depending on what stimuli are detected in these environmental scans, awakening may be initiated to better respond to potential threats. Based on phylogenetic predictions, human sleep on average is much shorter than expected and is characterized by a higher proportion of REM sleep compared to that of other primates [19]. It is hypothesized that humans evolved this shorter, more efficient sleep in part to minimize time that they are especially vulnerable to environmental threats when in deep, high arousal threshold sleep [19, 20].

The sentinel hypothesis further states that humans and other animals would have learned that sleep is only safe when other individuals in their group remain alert, acting as sentinels [16]. Hunter-gatherers exemplify patterns of human behavioral ecology in the absence of agriculture, therefore providing the closest approximation to ancestral sleep-wake patterns [21]. Samson and colleagues [8] found in a community of Hadza in Tanzania, throughout a 20-day study period, simultaneous sleep accounted for only 18 min in total observation—with a median of eight individuals awake throughout the nighttime period. In other words, between the time when the first person went to sleep and the last person awoke, one or more individuals was awake during 99.8% of sampled epochs. This observed group chronotype diversity (i.e. individual differences in sleep timing preference)—which is not planned or intentional—supports the idea that human sleep is shaped by the need to minimize risk while in a reduced state of alertness by selecting for group sentinel behavior [8].

### Evolutionary medicine perspectives on sleep

Evolutionary medicine proposes that modern human physiology and behavior reflect adaptations to selective pressures characteristic of our recent ancestral environments. However, some of these adaptations may now be mismatched with our environments and are implicated in adverse health outcomes [22]. Evolutionary mismatches refer to traits that were once adaptative but lose their adaptive benefits when the environment changes relatively rapidly, thus becoming maladaptive [23]. Remaining vigilant to potential threats in physical surroundings has likely been selected for over the course of human evolution. In only very recent human history has technological infrastructure and reinforced housing protected us from the numerous sources of threat in our environments, including other humans, animals, and inclement weather. However, our physiological responses to signs of potential threat such as noise, nighttime lighting, or social conflict may not have “caught up” to our relatively safer sleeping environments, meaning that harmless noise from other people, traffic, or animals may in part contribute to a mismatch scenario that leads to sleep disorders.

Is sentinel behavior a universal characteristic of human sleep patterns, or is it expressed differentially depending on environmental cues that signal the need for increased vigilance? The current study addresses this question by testing the sentinel hypothesis in nonindustrial Hadza [24] and Malagasy [9] samples and comparing their sleep to two samples from the United States. The first comparison is with participants from the Midlife in the United States (MIDUS) study, a longitudinal health and wellbeing dataset of majority non-Hispanic White (NHW), middle class Americans [25]. The second comparison is with participants in the Hispanic Community Health Study/Study of Latinos (HCHS/SOL) project comprised of individuals of Cuban, Dominican, Mexican, Puerto Rican, Central American, and South American background living in the United States [26].

In testing the sentinel hypothesis, we propose that sentinelization of sleep (e.g. multiple awakenings throughout the night) is a passive behavior that is still expressed in human sleep. We hypothesize that sentinel behavior is an evolved expression of flexible sleep patterns in humans that is more apparent in sleep environments where greater noise exposure leads to activation of a vigilance response, resulting in more sleep disruption. We therefore predict that sentinelization will be highest in Hadza and Malagasy samples due to their relatively less secure sleeping environments and corresponding exposure to stimuli that may prompt awakening such as noise, smoke from fires, and temperature fluctuations, and lowest in the two samples from the United States, reflecting sleep environments that are technologically buffered from environmental stimuli that may lead to a heightened threat response.

## METHODS

### Participants

Data were analyzed using a sample of 1,949 participants in total from Hadza hunter-gatherers [24], Malagasy small-scale agriculturalists [9], Hispanics from the Hispanic Community Health Study/Study of Latinos (HCHS/SOL) Sueño Ancillary Study, and NHWs from the Midlife in the United States (MIDUS) cohorts.

#### Hadza

A total of 33 (12 males, 21 females; mean age = 34.62) Hadza participants were recruited between June 2015 and February 2016. Hadza are equatorial hunter-gatherers from near Lake Eyasi in Northern Tanzania. Their diet consists of hunted game animals, birds, and honey, as well as gathered fruits, nuts, seeds, tubers, and legumes. There is a pronounced sexual division of labor, with men primarily hunting and women primarily gathering [8]. The study protocol followed the Duke University and the University of Nevada, Las Vegas Institutional Review Boards for human subjects research. Verbal informed consent was obtained from all participants, and research was approved by the Tanzanian Commission for Science and Technology (COSTECH) and the Tanzanian National Institute for Medical Research (NIMR).

#### Malagasy

Thirty-eight Malagasy participants (19 males, 19 females; mean age = 41.07 years) were recruited from Madagascar in three field seasons (July–August 2015 and 2016 and November–December 2017). Malagasy participants are from Mandena, a rural community in the northeastern part of the country. The community has no electric infrastructure and depends on small-scale, subsistence agriculture, but economic development has prompted increased economic shifts away from this pattern of subsistence [9]. Written informed consent was obtained from all subjects, and followed the protocol outlined by the Duke University Institutional Review Board for human subjects research.

#### Non-Hispanic White

NHW, middle-class, American participants (*n* = 347; 160 males, 187 females; mean age = 52.67 years) were included from the MIDUS longitudinal cohort. The project was started in 1995–1996 by the MacArthur Foundation Research Network on Successful Midlife Development, with the aim of explaining how behavioral, psychological, and social factors affect age-related health and wellbeing. The project now consists of multiple related studies, including data from over 10,000 individuals between the ages of 24 and 74 living in the United States. Participants throughout the country were interviewed by phone and self-administered questionnaire, answering questions related to demographic variables, health, employment, and psychological factors [25].

Sleep data come from the MIDUS II and MIDUS Refresher datasets. MIDUS II was conducted in 2004 and included follow-up of the data collected in MIDUS I as well as cognitive, neurological, and comprehensive biomarker assessments, and sleep data from subsamples of respondents [27]. MIDUS Refresher data were collected between 2012 and 2016 [28]. We excluded participants taking sleep medications more than once or twice a week and those with diagnosed sleep disorders (e.g. insomnia). The protocol for data collection was approved by the Education and Social/Behavioral Sciences and the Health Sciences Institutional Review Boards at the University of Wisconsin-Madison.

#### Hispanic

Hispanic participants include 1,531 individuals (566 males, 965 females; mean age = 46.14 years) from the Sueño ancillary study of the Hispanic Community Health Study/Study of Latinos (HCHS/SOL). The HCHS/SOL study investigates health outcomes in self-identified Hispanic/Latinx individuals randomly selected from four communities in the United States (Bronx, New York; Chicago, Illinois; Miami, Florida; San Diego, California). The aim of the study is to examine cardiovascular disease risk factors among American Hispanic/Latinx individuals and to assess the role of social factors related to disease risk. Data were originally collected from 15,079 participants between March 2008 and June 2011 and included a physical exam, blood samples, dental exam, hearing test, pulmonary function, physical activity assessment, and questionnaire data. Questionnaire data yielded information related to demographic details, socioeconomic status, and health history and behavior [26].

Sleep data were obtained from the Sueño ancillary study, which recruited 2,252 total individuals within 30 months of their baseline HCHS/SOL examinations. Sleep monitoring was performed between December 2010 and December 2013. Only participants negative for narcolepsy and severe obstructive sleep apnea (AHI ≥ 50/h), and not using nocturnal positive airway pressure therapy, were recruited [29]. For our analysis, we further excluded participants taking sleep medications more than once per week and those with diagnosed sleep disorders (e.g. insomnia). Data collection followed protocols at the institutional review boards at the field centers and coordinating center institutions.

### Equipment and protocol

All sleep data were obtained with accelerometry using wrist-worn actigraphs. Actigraphs are wearable devices that provide non-invasive measures of sleep and wake activity. Actigraphs are validated to a high degree of reliability against polysomnography with the added advantage of enabling data collection of sleep measures in ambulatory participants within their natural sleeping environments [30, 31]. Activity is measured with a built-in, high-sensitivity accelerometer that logs data over a user-defined interval and translates movement into a binary sleep-wake determination.

#### Nonindustrial groups

Hadza and Malagasy participants were informed of the study objectives by local translators, and instructed to wear the watches for the duration of the study. Participants were asked to press an event marker button, which helps in actigraphy scoring for identifying sleep times, wake times, and naps. Sleep was measured in both groups using the CamNtech MotionWatch 8 actigraph (CamNtech, Cambridge, United Kingdom), collected in 60-second intervals. Data were then scored using the CamNtech MotionWare 1.1.15 program [8, 9]. Total nights of actigraphy range from 4-20 nights for the Malagasy sample and 3-20 for the Hadza sample.

#### Industrial groups

MIDUS sleep data were obtained using the Mini Mitter Actiwatch-64 (Philips Healthcare, Amsterdam, Netherlands) activity monitor, worn by participants for seven consecutive days. Rest, sleep, and active period data generated by the Actiwatch were used to generate summary statistics. The Actiwatch-64 uses a built-in sensor to detect movement during a 30-s interval. Data were processed using Actiware Software to generate summary statistics of the sleep period for a given day [32].

Biometric sleep data were obtained from HCHS/SOL Sueño participants with the Actiwatch Spectrum actigraph (Philips Respironics, Murrysville, Pennsylvania, United States). Participants were instructed to wear the watches for the seven day duration of the study, which measured sleep-wake activity in 30-s epochs [33]. Upon completion of the study, data were sent to a central reading center where they were scored using a standardized method to evaluate and clean data for analysis. Epoch by epoch sleep/wake status was then generated by the Actiware 5.59 algorithm [29].

### Data analysis

The main sleep variable of interest is average time in hours spent awake after sleep onset across all bouts of awakening (wake after sleep onset; WASO). We chose WASO as the closest proxy for the brief awakenings discussed in the sentinel hypothesis [16]. We also present average time in bed and average hours of sleep per sleep period (sleep duration) to better contextualize general sleep patterns in the comparative populations.

To test the sentinel hypothesis, we modeled data using linear regression. We modeled sleep variables of interest as a function of group membership (i.e. NHW, Hispanic, Hadza, Malagasy) and controlled for sex and age. Models were built using the R programming language [34]. In addition to modeling variation in WASO, we also modeled predictors of time in bed and sleep duration. Previous studies have reported that females may experience longer, higher quality sleep on average [35, 36], so we included an interaction of sex and group to investigate whether sex has a differential effect on sentinelized sleep patterns within each group (e.g. WASO ~ Group*Sex + Age).

### Supplementary analysis

We performed supplemental regression analysis of WASO within the Hispanic sample to further investigate the role of perceived environmental threat on sleep disruption. We used available variables from the HCHS/SOL dataset that asked about neighborhood safety and racism. We primarily focused on the Neighborhood Safety survey items in which participants were asked to “Think about your neighborhood as a whole, then please choose the response for each of the following to show how much a problem each one is in your neighborhood”. We focused on the problems of violence and excessive noise, which were rated on a scale of 1 = Very Serious Problem to 4 = Not Really a Problem. We also used Racism/Discrimination Score, in which participants were asked about their lifetime experiences with racial discrimination, with responses on a scale of 1 to 5, with 1 being never, 3 sometimes, and 5 very often. For neighborhood variables, we included the 1,528 participants with responses, and we built a linear regression model to predict WASO as a function of neighborhood violence and excessive noise, controlling for age and sex.

## RESULTS

Descriptive statistics of demographic and sleep characteristics by group are presented in Table 1. Wake after sleep onset (WASO) is highest in Hadza and Malagasy (2.44 and 1.93 h, respectively), and lowest for NHW respondents (0.69 h). At 0.86 h of WASO, Hispanic participants demonstrate less time awake during sleep periods than Hadza and Malagasy, but more than NHW participants. Hadza participants have the longest average time in bed (9.16 h), and NHW participants have the shortest average time in bed (7.14 h). Hispanic participants have the longest average sleep duration (6.67 h), while Hadza have the shortest average sleep duration (6.24 h).

**Table 1.** Sample characteristics by group, presented as mean (standard deviation) or *N* (percentage)

	Non-Hispanic White	Hispanic	Hadza	Malagasy
	(*n* = 347)	(*n* = 1,531)	(*n* = 33)	(*n* = 38)
Sex				
Male	160 (46%)	566 (37%)	12 (36%)	19 (50%)
Female	187 (54%)	965 (63%)	21 (64%)	19 (50%)
Age, years	52.67 (12.87)	46.14 (11.91)	34.62 (13.32)	41.07 (13.19)
WASO (h)	0.69 (0.31)	0.86 (0.41)	2.44 (0.66)	1.93 (0.50)
Time in bed (h)	7.14 (1.02)	7.60 (1.11)	9.16 (0.72)	9.05 (0.91)
Sleep duration (h)	6.44 (1.01)	6.67 (1.02)	6.24 (0.72)	6.57 (0.95)

### Group membership as a predictor of WASO

**Figure 1.**
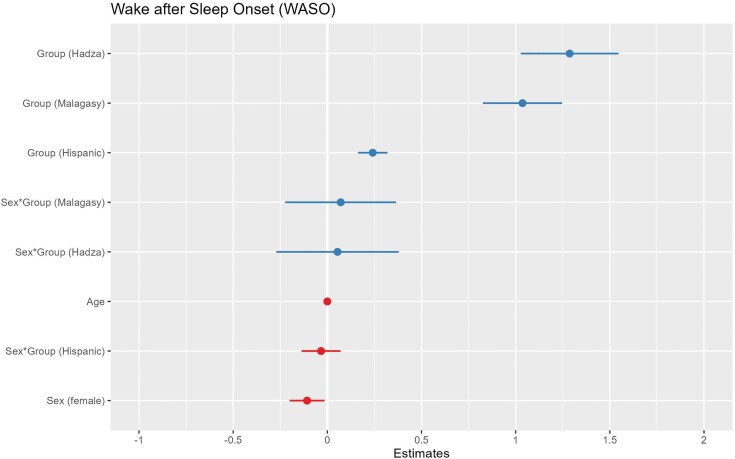
A coefficient plot for wake after sleep onset (WASO). Hadza, Malagasy, and Hispanic group membership predicts significantly greater WASO than non-Hispanic White group membership. Female sex predicts significantly less WASO. The plotted lines show the 95% confidence intervals of each predictor variable. Continuous predictor variables were scaled for comparability of coefficients.

To test the sentinel hypothesis, we fitted a linear model predicting WASO as a function of group membership. Our results show that WASO varies significantly by group. Hadza (Estimate=1.287, SE = 0.132, CI = 1.027 to 1.546), Malagasy (Estimate = 1.036, SE = 0.107, CI = 0.826 to 1.247), and Hispanic (Estimate = 0.241, SE = 0.040, CI = 0.163 to 0.319) group membership predicts significantly greater WASO compared to NHW. Female sex (Estimate = −0.108, SE = 0.047, CI = −0.200 to −0.015) predicts lower WASO values compared to males (see Fig. 1).

### Group membership as a predictor of time in bed and sleep duration

**Figure 2.**
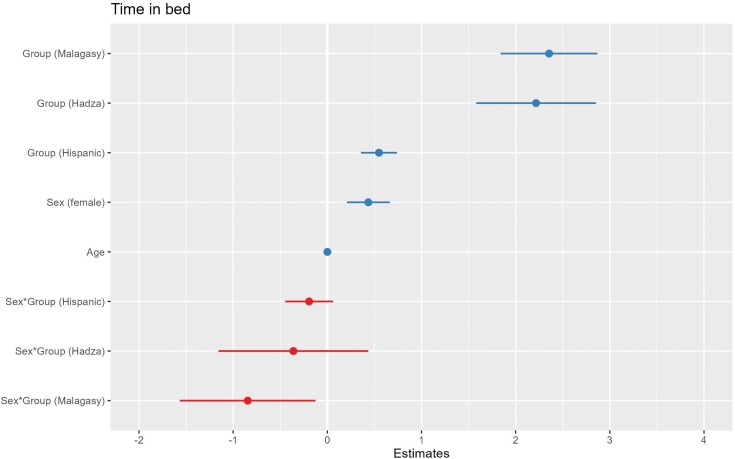
A coefficient plot for time in bed. Hadza, Malagasy, and Hispanic group membership predicts significantly longer time in bed than non-Hispanic White group membership. Female sex also predicts longer time in bed. An interaction of sex and Malagasy group membership predicts shorter time in bed. The plotted lines show the 95% confidence intervals of each predictor variable. Continuous predictor variables were scaled for comparability of coefficients.

**Figure 3.**
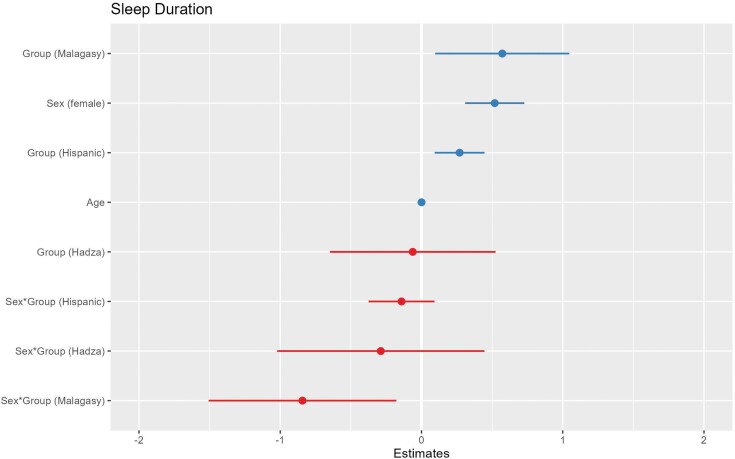
A coefficient plot for sleep duration. Hadza and Malagasy group membership predicts significantly shorter sleep duration than non-Hispanic White group membership, while Hispanic group membership and female sex predict longer duration. The plotted lines show the 95% confidence intervals of each predictor variable. Continuous predictor variables were scaled for comparability of coefficients.

Compared to the NHW reference, membership in all groups predicts significantly longer time in bed (Hadza: Estimate = 2.216, SE = 0.324, CI = 1.581 to 2.852; Hispanic: Estimate = 0.549, SE = 0.0978, CI = 0.357 to 0.740; Malagasy: Estimate = 2.355, SE = 0.262, CI = 1.840 to 2.869). Female sex also predicts longer time in bed (Estimate = 0.436, SE = 0.116, CI = 0.209 to 0.663) (Fig. 2). Hispanic (Estimate = 0.270, SE = 0.090, CI = 0.093 to 0.447) and Malagasy (Estimate = 0.572, SE = 0.242, CI = 0.097 to 1.047) group membership and female sex (Estimate = 0.519, SE = 0.107, CI = 0.309 to 0.728) predict significantly longer sleep duration (Fig. 3).

### Interaction of sex and group membership

Descriptive statistics of sleep averages by sex within each group are presented in Table S1. WASO, time in bed, and sleep duration significantly vary by sex in NHW and Hispanic groups, but no sex differences are significant in Hadza or Malagasy. In modeling an interaction of sex and group membership, there were no significant interactions between sex and group in predicting WASO (sex*Hadza: Estimate = 0.054, SE = 0.166, CI = −0.271 to 0.379; sex*Malagasy: Estimate = 0.071, SE = 0.150, CI = −0.224 to 0.365; sex*Hispanic: Estimate = −0.033, SE = 0.053, CI = −0.137 to 0.070). In the time in bed and sleep duration interaction models, an interaction of female sex and Malagasy group membership predicts shorter time in bed (Estimate = −0.845, SE = 0.367, CI = −1.566 to −0.125) and sleep duration (Estimate = −0.843, SE = 0.339, CI = −1.507 to −0.178). Full results of regression models are presented in Table 2.

**Table 2.** Results from linear regression model predicting WASO, time in bed, and sleep duration, with an interaction between sex and group

	WASO		Time in bed		Sleep duration	
	Estimate (SE)	95% CI	Estimate (SE)	95% CI	Estimate (SE)	95% CI
Age	−0.0002	−0.002, 0.001	0.0004	−0.004, 0.004	0.0002	−0.003, 0.004
	(0.001)		(0.002)		(0.002)	
Sex (female)	−0.108^*^	−0.200, −0.015	0.436^***^	0.209, 0.663	0.519^***^	0.309, 0.728
	(0.047)		(0.116)		(0.107)	
Group (Hadza)	1.287^***^	1.027, 1.546	2.216^***^	1.581, 2.852	−0.062	−0.648, 0.524
	(0.132)		(0.324)		(0.299)	
Group (Hispanic)	0.241^***^	0.163, 0.319	0.549^***^	0.357, 0.740	0.270^**^	0.093, 0.447
	(0.040)		(0.098)		(0.090)	
Group (Malagasy)	1.036^***^	0.826, 1.247	2.355^***^	1.840, 2.869	0.572^*^	0.0974, 1.047
	(0.107)		(0.262)		(0.242)	
Sex (female)*Group (Hadza)	0.054	−0.271, 0.379	−0.360	−1.156, 0.435	−0.288	−1.022, 0.446
	(0.166)		(0.406)		(0.374)	
Sex (female)*Group (Hispanic)	−0.033	−0.137, 0.070	−0.193	−0.446, 0.060	−0.141	−0.375, 0.092
	(0.053)		(0.129)		(0.119)	
Sex (female)*Group (Malagasy)	0.071	−0.224, 0.365	−0.845^*^	−1.566, −0.125	−0.843^*^	−1.507, −0.178
	(0.150)		(0.367)		(0.339)	

Non-Hispanic White is the reference group and male is the reference sex.

Significance codes:

^*^ *P* < 0.05;

^**^ *P* < 0.01;

^***^ *P* < 0.001.

### Neighborhood safety and racism predictors of WASO in the Hispanic group

We found that perceived neighborhood violence (Estimate = −0.030, SE = 0.013, CI = −0.056 to −0.005), but not noise (Estimate = −0.004, SE = 0.013, CI = −0.030 to 0.022), is a significant predictor of increased WASO. Controlling for age and sex, higher racism scores are only marginally significant (Estimate = 0.029, SE = 0.015, CI = −0.001 to 0.058). Supplementary regression results are presented in Table S2 and Table S3.

## DISCUSSION

In modeling WASO as a function of group membership, we found that hunter-gatherer Hadza and small-scale agriculturalist Malagasy samples exhibit evidence of greater WASO as a proxy of more sentinel behavior than either of the samples from the United States. Findings of significantly greater WASO demonstrate that the nonindustrial populations are waking more throughout the night. These results are consistent with previous literature suggesting increased vigilance expressed in the sleep of small-scale groups, which is consistent with our hypothesis that groups sleeping in louder, less reinforced housing experience sleep disruption as a response to external cues. Previous results have demonstrated sentinelized sleep in Hadza participants, whose sleep patterns are characterized by a very narrow window during which all members of the group synchronously sleep [8]. This behavior is not planned or intentional, but is instead hypothesized to be a passive behavior that has remained part of human sleep ecology. The current study’s comparison of a Hadza population with a similarly nonindustrial community of Malagasy further supports the hypothesis that sentinel behavior may still be an important aspect of human sleep ecology, stemming from the evolved need to respond to signals during the sleep period.

Interestingly, nightly sleep duration does not appear to be as differentially expressed across socio-ecological contexts as is WASO. Hispanic and Malagasy participants sleep significantly longer than NHW participants, so we do not see clear evidence for industrialization being strongly associated with sleep duration. This consistency across groups suggests that sleep duration could be a more highly regulated biological process than is WASO, which we propose is more sensitive to external environmental stimuli. We found that groups exhibiting higher WASO also have longer time in bed. It is possible that longer time in bed allows more awakenings throughout the night while still achieving the required amount of sleep. It is also possible that greater WASO necessitates longer time in bed to make up for the sleep time that is lost during nighttime awakenings. Both possibilities speak to a relationship between time in bed and WASO, and also suggest flexibility in the mechanisms by which sufficient nightly sleep duration is achieved in diverse socio-ecological settings.

### Sleep and technological infrastructure

**Figure 4.**
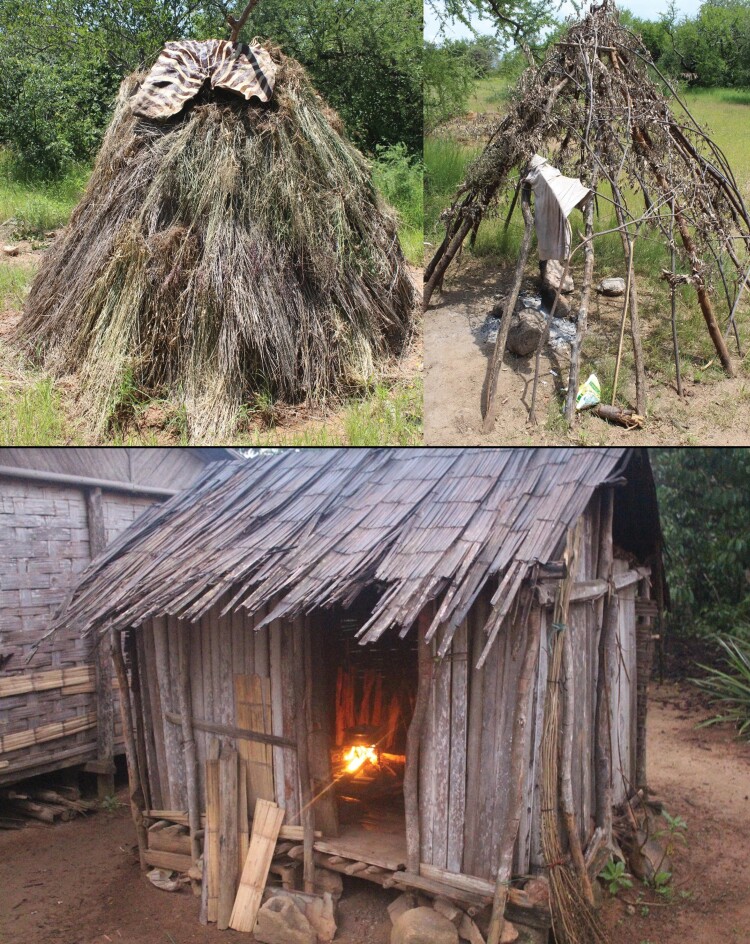
Top: Hadza home constructed from branches stuck into the ground (right) and then stuffed with grasses (left). Bottom: Malagasy home constructed from wood. Participant consent for photographs was given to DRS.

Industrial sleeping environments are characterized by varying degrees of continuous technological buffers to the environment (e.g. climate and noise-controlled sleep sites), which protect against noise during the night that could activate a threat response and lead to awakening. It has been found that humans process and evaluate auditory stimuli during sleep [18], so quieter sleeping environments could help to minimize awakening responses. Although industrialized sleeping environments are often characterized by nighttime light and noise from traffic and other people—particularly in urban settings—dwellings are much more insulated and buffered from outside disturbance than the sleeping sites of Hadza and Malagasy (Fig. 4), which are much more exposed to environmental noise, light, and activity throughout the night. Hadza huts are often situated within earshot of multiple other huts, and noise is associated with wakefulness in Hadza camps, likely attributed to campfire activity, ritual ceremonies, and sexual activity [24]. The sleep-disrupting effects of these activities are more pronounced given the low-insulation of Hadza and Malagasy sleeping environments, where sound carries more than it would in a house or apartment in the United States. Furthermore, we hypothesize that unlike the impersonal sounds of nighttime city traffic or neighborhood activity in the United States, the sounds coming from a Hadza or Malagasy camp are likely related to kin or group members and warrant more attention. We propose that these factors may all contribute to activation of vigilance responses in the form of increased WASO.

Despite the comfort and safety afforded by industrial infrastructure, the regular use of artificial lights after sunset and exposure to noise pollution that are characteristic of urban settings are often blamed for contributing to poor sleep outcomes [37]. Indeed, higher rates of insomnia and sleep disturbance have been reported in developed countries and metropolitan populations [38, 39]. In small-scale subsistence societies with and without access to artificial lighting, electric infrastructure is associated with shorter habitual sleep duration and later bedtime (e.g [40–42].). The results of the current study are in contrast to these findings, as sleep disruption is greater in nonindustrial populations, with greater time spent awake during the night. These findings are consistent with previous results from nonindustrial populations that have reported lower efficiency and/or shorter sleep duration compared to industrial populations [9, 42, 43]. Overall, our results suggest that the relationship between industrialization and sleep is complex, but that some aspects of technological infrastructure in industrial populations may facilitate feelings of safety that lead to better sleep.

### Sex, gender roles, and sleep patterns

Female sex was a significant predictor of lower WASO and longer time in bed and sleep duration in our regression models. Our results on sleep duration are consistent with previous literature demonstrating that women experience longer sleep when using objective analysis methods (e.g. actigraphy, polysomnography) [35, 36]. Interestingly, when examined by group membership, sex differences were only significant in NHW and Hispanic participants, and not Hadza and Malagasy (see Table S1). It is possible that higher occurrences of co-sleeping and young children in the sleeping environment may disrupt females and males similarly in the nonindustrial groups, so that sex differences in WASO are minimized. In other words, a crying infant in a relatively more crowded, less noise-insulated hut is more likely to wake everyone—females and males alike—in both the same hut and even nearby dwellings. In the full regression models, the interaction of sex and Malagasy group membership was significant in predicting significantly lower sleep duration, suggesting the possibility that culturally constructed gender roles (e.g. childcare, economic responsibilities) may have an influence on the observed sleep patterns.

To date, the majority of research conducted on sex differences in sleep duration and quality are based in Western, industrial contexts, so it is unsurprising that our findings of sleep in the United States populations follow these norms; yet, in small-scale nonindustrial populations they do not. It is possible that gendered labor roles, subsistence strategy, and social norms contribute to these differences by population. For instance, Hadza activities demonstrate strong gendered division of labor, with men the primary hunters and women the primary gatherers [44]. Hadza women breastfeed their infants and children on-demand up to around age two, and breastfeeding women are found to have earlier wake times and lower activity during the day compared to men and non-breastfeeding women [45]. Combining ethnographic and qualitative data on sleep in nonindustrial settings will clarify our understanding of the relative contribution of biocultural factors in shaping sex and gender-based sleep patterns. Overall, while we found that there is more sentinel behavior (i.e. WASO) in males than females, this difference is only significant in the NHW and Hispanic samples, and there is no significant interaction effect of group and sex in predicting WASO in the regression models. Variation in the effect of perceived threat on sentinel behavior should be explored in future work to assess whether there are sex differences in sentinel behavior that are not captured by our analysis.

### Sleep health disparities, social stigma, and discrimination

While our prediction that industrial groups would experience less WASO compared to nonindustrial groups was supported, we found that Hispanic participants have significantly greater WASO than NHW participants. We speculated that this pattern could have to do in part with the effects of social marginalization of individuals of Hispanic heritage in the United States. We investigated this hypothesis through performing supplemental regression analysis of WASO and perceived neighborhood safety and racism within the Hispanic sample. These supplementary analyses, particularly on perceived neighborhood violence and noise, support the idea that in the analyzed sample of Hispanics from the United States, perceived threats of violence are a more significant disruptor of sleep than noise. These findings are consistent with previous research. Neighborhood and home safety have been reported to predict sleep outcomes [46, 47], including in previous analysis using the HCHS/SOL sample in which lower perceived neighborhood safety was found to significantly increase odds of short sleep duration [48].

Surprisingly, Racism/Discrimination Score did not significantly predict WASO in our supplementary analysis. It is possible that this measure does not fully capture the lived experience of participants, as the measure asks about lifetime discrimination experiences and may not reflect actual living and sleeping environments that participants are currently in. Nevertheless, Racism/Discrimination Score was marginally significant in predicting more WASO, and it still warrants discussion given the strong previous evidence for the negative effects of racism and stigma on sleep health. Systemic racism in the United States is strongly implicated in driving widespread healthcare inequalities for Americans of color [49], and racial and ethnic sleep disparities are well documented. Alcántara and colleagues [50] found that discrimination based on ethnicity and acculturation stress were associated with increased daytime sleepiness [50]. Numerous studies in the United States have found that compared to NHW participants, Black participants demonstrate shorter sleep duration and lower sleep efficiency compared to NHW Americans [51–54], and Black Americans have been found to be more likely to experience sleep insufficiency [55]. A large study using nationally-representative data from the Behavioral Risk Factor Surveillance System found that Black Americans were significantly more likely to report fewer than 7 h of sleep per night [56]. These sleep health inequities are linked to experiences of racism. In a recent study of self-reported health in 422 African American women, personal, direct experiences with racism—particularly violent experiences—were found to be associated with poor self-reported sleep quality [57]. Given the serious health problems associated with insufficient sleep, racism and discrimination are important for considering sleep outcomes within the broader discussion of health inequities.

Our results suggest that health disparities related to neighborhood safety may in part be related to our evolved vigilance to lethal and non-lethal threats in our surroundings, whether real or perceived. Disrupted sleep may reflect a response consistent with the “smoke detector principle,” which proposes that defense mechanisms that cost less than the potential threat they protect against will often trigger false alarms [58]. In other words, the fitness costs of brief sleep disruptions would have been less than even a small risk of predation in our evolutionary history. From a fitness perspective, overreactions in the awakening response to potential danger would have likely been favorable to underreacting and sustaining serious injury or death. While acute sleep disruption may have increased immediate survival in human evolutionary history, this evolved alertness may now be contributing to health concerns such as increasing reports of chronic sleep disruption and insomnia [20, 59], particularly in communities who experience elevated social stigma.

Consistent with an evolutionary medicine perspective, rather than viewing observed sleep disparities as stemming solely from sleep pathologies, we propose that disrupted sleep may in part be related to evolved vigilance mechanisms that were adaptive in our ancestral environment and that current conditions of discrimination and inequitable housing and neighborhood quality arouse these ancient mechanisms to the detriment of health and wellbeing. Evolutionary medicine explains human response mechanisms that have become maladaptive in our safer modern environments, and also indicates where it may be appropriate to minimize our anxiety, stress, and pain responses without a corresponding decrease in fitness [58]. Although our results suggest that in some cases it may be appropriate to classify modern sleep disturbances as overreactions to our relatively safer environments (e.g. harmless noise from city traffic), we should also be cognizant that our safe modern environments are not equally safe for everyone. Social stigma and discrimination should be considered for their real effects on the safety of sleeping and living environments. Therefore, while improving physical sleeping environments would go some way toward redressing sleep health disparities, it must be combined with addressing racial and ethnic social stigma in order to minimize social threat as well. This would likely improve not only sleep health, but also the general health outcomes that sleep underpins.

### Limitations

There are potential limitations to our findings. First, WASO is most likely driven by a complex interaction of different types of nighttime disturbances, including noise from animals, other people, children crying, co-sleeping, breastfeeding and childcare responsibilities, sexual activity, or other factors that are unrelated to vigilance mechanisms. While our results are consistent with the idea that increased alertness during sleep may have been adaptive in our evolutionary history, it is impossible to disentangle the numerous socio-ecological factors—some unrelated to threat and vigilance—that could be contributing to differential expressions of sleep sentinelization. Further research should also model reported perception of safety as a predictor of WASO, which was not available in the Hadza and Malagasy datasets at the time of our analysis.

Second, our regression analyses did not account for co-sleeping. Previous work with Hadza groups has suggested that number of co-sleepers (but not breastfeeding) is associated with shorter sleep duration and lower sleep quality [45]. To the best of our knowledge, co-sleeping data are not available in the HCHS/SOL dataset, so could not be included in our main regression models. However, we included co-sleeping as a covariate in our exploratory analysis (not shown) of predictors of WASO in Hadza, Malagasy, and the MIDUS II cohort, and found that co-sleeping was not significant. Despite this non-significant finding, we believe that co-sleeping, bedsharing, and childcare may still be playing a greater role in driving WASO than our data are able to show, particularly in hunter-gatherer groups such as the Hadza where women’s activity and sleep patterns are affected by long-term, on-demand breastfeeding of infants and children [45]. It is also worth noting that co-sleeping and/or the presence of infants can be related to WASO apart from nursing or other activities. Sentinelized sleep patterns (i.e. greater WASO) may be exaggerated in the presence of others—particularly vulnerable infants and children—as a way of not only ensuring one’s own safety, but also that of co-sleepers and dependents.

Third, while the use of diverse datasets provided valuable cross-cultural comparisons in our test of the sentinel hypothesis, there are methodological limitations. Sleep data were generated from CamNtech MotionWatch 8 actigraphs (Hadza and Malagasy), Mini Mitter Actiwatch-64 activity monitors (MIDUS), and Actiwatch Spectrum actigraphs (HCHS/SOL). Similarly, sleep data scoring and optimization software corresponded to their respective accelerometers. Data cleaning and optimization were performed by different individuals trained in separate protocols, introducing potential inconsistencies and rater bias. MIDUS data were collected in 30-s intervals, unlike the other three groups in which data were collected in 60-s intervals. Differences in actigraphy results generated from 60-s intervals vs 30-s intervals have been previously found to be insignificant [60], and we addressed the other limitations to the best of our ability by choosing sleep variables that were generated with the most consistent protocols, but they should be considered in the comparability of sleep patterns across the four samples.

## CONCLUSIONS

In summary, this work presents support for the idea that sleep disruption is more common in socio-ecological environments with more frequent night disturbances, a behavior that may have conferred a past adaptive advantage. Increased WASO is observed in small-scale, nonindustrial samples compared to industrial samples from the United States, suggesting that sentinelization and a high degree of nighttime awakenings may have been characteristic of ancestral humans’ sleep. The reduced WASO in the United States suggests that quieter, secure housing can buffer against environmental stimuli that lead to awakenings, and reduce sentinel sleep behavior that could be contributing to disrupted sleep. However, our finding that Hispanic participants experience more awakenings throughout the night, which is associated with perceived neighborhood violence, should encourage further research into the sources of sleep disruption in minority communities in the United States, which could reflect the adverse health effects of neighborhood inequities and health disparities stemming from social marginalization. While sentinelized sleep patterns could in part stem from a behavior that increased immediate survival of our ancestors through increased vigilance, it may now be contributing to insufficient sleep and maintenance of an evolved fear response, which may be associated with increased insomnia [59]. Critically, with increasing reports of sleep insufficiency that are often described as a sleep loss epidemic [61, 62], our results contribute to the field of evolutionary medicine by increasing our understanding of the possible evolutionary explanations for sleep disturbances. Understanding and addressing the environmental and social factors that lead to heightened vigilance during sleep should be prioritized to better understand and improve sleep and overall health.

## Supplementary Material

eoac039_suppl_Supplementary_Material
