# Supplementary material for: Sound reasons for unsound sleep: Comparative support for the sentinel hypothesis in industrial and nonindustrial groups
Source: Evol Med Public Health. 2022 Nov 22;11(1):53–66. doi: 10.1093/emph/eoac039 (PMC10024786; doi:10.1093/emph/eoac039)
Supplement: eoac039_suppl_Supplementary_Material [file eoac039_suppl_supplementary_material.pdf]

Supplementary Information

Table S1. Sample characteristics by sex within each group, presented as mean (standard deviation) and t-test results.

	**Non-Hispanic White**			**Hispanic**			**Hadza**			**Malagasy**		
	Male	Female		Male	Female		Male	Female		Male	Female	
Wake after sleep onset (hours)	0.74	0.65	*	0.95	0.81	***	2.53	2.39	ns	1.98	1.87	ns
Time in bed (hours)	6.90	7.34	***	7.45	7.69	***	9.11	9.19	ns	9.25	8.84	ns
Sleep duration (hours)	6.16	6.68	***	6.43	6.81	***	6.10	6.33	ns	6.73	6.41	ns

Note: Significance is denoted with stars relative to t-test comparisons between males and females.

Significance codes: * $p < 0.05$; ** $p < 0.01$; *** $p < 0.001$

Table S2. Results from linear regression model predicting wake after sleep onset (WASO) in the HCHS/SOL sample, including neighborhood noise and crime covariates. Male is the reference sex. Model includes 1,528 participants from HCHS/SOL.

	**WASO**	
	Estimate	*95% CI*
	(SE)	
Age	-0.008	-0.030, 0.015
	(0.011)	
Sex (female)	-0.144***	-0.191, -0.098
	(0.024)	
Neighborhood Noise	-0.004	-0.030, 0.022
	(0.013)	
Neighborhood Violence	-0.030*	-0.056, -0.005
	(0.013)	

Significance codes: * $p < 0.05$; ** $p < 0.01$; *** $p < 0.001$

Table S3. Results from linear regression model predicting wake after sleep onset (WASO) in the HCHS/SOL sample, including racism scale and neighborhood noise and violence as covariates. Male is the reference sex. Model includes 876 participants from HCHS/SOL.

	**WASO**	
	Estimate	*95% CI*
	(SE)	
Age	-0.021	-0.050, 0.009
	(0.015)	
Sex (female)	-0.129***	-0.190, -0.067
	(0.031)	
Racism/Discrimination Score	0.029	-0.001, 0.058
	(0.015)	

Significance codes: * $p < 0.05$; ** $p < 0.01$; *** $p < 0.001$
